# RNAi dependent epigenetic marks on a geminivirus promoter

**DOI:** 10.1186/1743-422X-3-5

**Published:** 2006-01-30

**Authors:** Afzal Muhammad Dogar

**Affiliations:** 1 Swiss Institute for Experimental Cancer Research (ISREC), Ch. des Boveresses 155, CH-1066 Epalinges, Switzerland

## Abstract

*Nicotiana benthamiana *plants were stably transformed with an intron-spliced dsRNA producing construct cognate to bidirectional promoter of African cassava mosaic geminivirus (ACMV) DNA A. Transgenic lines expressed multiple siRNAs species upon ACMV infection. The *de novo *DNA methylation and an increased proportion of histone H3 Lysine-9 methylation (H3K9) at intergenic region (IGR) of ACMV DNA A were observed.

## Introduction

In plants RNA interference or post transcriptional gene silencing (PTGS) acts as a natural anti-viral defense system for neutralizing pathogenic nucleic acids either through a change in RNA stability in the cytoplasm or through mechanisms that use the RNA itself to induce methylation and silencing of homologous nuclear genes [1]. In addition there are more than one Dicer and Argonaute proteins in plants e.g. Arabidopsis genome encodes four Dicers [2] and ten Argonate orthologs [3]. Geminiviruses are single-stranded circular DNA viruses that cause economically significant diseases in a wide range of crop plants worldwide [4]. They replicate in the plant cell nuclei through transcription and replication competent double stranded DNA intermediate, which is packed into nucleosomes from host cells [5]. In plants, some geminivirus-host interactions naturally lead to host recovery e.g. natural recovery response induced by ACMV-infected *N. benthamiana *and cassava involves siRNAs originating from geminivirus genome [6]. However the affect of siRNAs at the virus genome remained to be seen. Here I show multiple siRNAs species in transgenic plants direct the methylation of ACMV DNA A as well as the methylation at lysine-9 residues of histone H3 wrapping the promoter region in the virus genome.

## The study

A 360 nucleotides fragment corresponding to the intergenic region of ACMV DNA A (GenBank: NC_001467) was cloned in sense and anti-sense orientation interrupted with a synthetic plant intron. The left arm *Kpn*I-*Cla*I and right arm *Xho*I-*Bam*I fragments were PCR amplifed and cloned into *dsproA VMYMV- int *vector [7]. The *Eco*RI-*Xba*I fragment of this vector was cloned into pCambia 1300. The following primers used for amplification the left arm (*Kpn*I F: GGTACCAATCTCAACTAGAGACACTCTTGA) and (ClaI R: ATCGATGCACAAATATTTAATTGCCAG), and the right arm (*Xho*I F: CTCGACGCAGTTTATAAATTAACGGGTC) and (*Bam*HI R: GGATCCAATGAGTTGATCTCTGTGAGAACT). The resulting binary construct was introduced into *Agrobacterium tumefaciens *LBA4404 by electroporation with a Gene Pulser apparatus (Bio-Rad). Seeds of wild type *Nicotina benthamiana *were grown on MS media at 25 to 27°C under artificial light (150 μmol s-1 m-2) for 16 h per day. Transgenic shoots were selected on Hygromycin at a concentration of 250 μg ml-1 and grown at 25 to 27°C under artificial light (150 μmol s-1 m-2) for 16 h per day. Seeds of T1 lines were grown on MS and two weeks old seedlings were infected with the infectious clones of ACMV Kenyan strain DNA A and ACMV Cameroon strain DNA B (GenBank: AF112353) using Bio Rad particle delivery system as previously described [8]. The siRNA isolation and analysis was performed as described previously [2]. The DNA probe used for siRNA northern hybridization (AGGGGCCAACCGTATAATATTACCC) corresponds to the Nona-nucleotide sequence within ACMV DNA A. Total DNA was extracted from different plant lines. 10 to 15 μg of DNA samples were digested overnight with *Sau*96 I. Resolved by electrophoresis on 0.8 to 1.0 % agarose gel and transfered overnight to a Hybond-N membrane. Southern hybridization was carried out with an ACMV DNA A promoter specific DNA probe labeled with digoxigenin (DIG) (Boehringer Mannheim Biochemicals) as described by manufacturers. The Chromatin immunoprecipitation (ChIP) was performed as described previously [9]. Anti-dimethyl-histone H3 [Lys9] #07–212 and anti- dimethyl-histone H3 [Lys4] #07–030 were purchased from Upstate Biotechnology. Each of the immunoprecipitation was performed at least three independent times. For each PCR reaction 2 μl of each immunoprecipiate used to amplify of the viral DNA and endogenous control. All PCR reactions were done in 25 μl volume, starting with 5 min at 96°C, followed by 30 cycles of 94°C (15 s), 57°C (30 s), and 72°C (5 min). The PCR reactions were analysed by electrophoresis on a 2% agarose gel. The primer pairs used were ACMV DNA A F: CTCAACTAGAGACACTCTTGA and R: CACAAATATTTAATTGCCAG, Tnt-retroposon (GenBank: X13777) F: CATTGGTTCTAAAGGATGTGCGGC and R: GAAATCTCATCTTGTGCCGCGTTC.

## Results and conclusion

**Figure 1:**
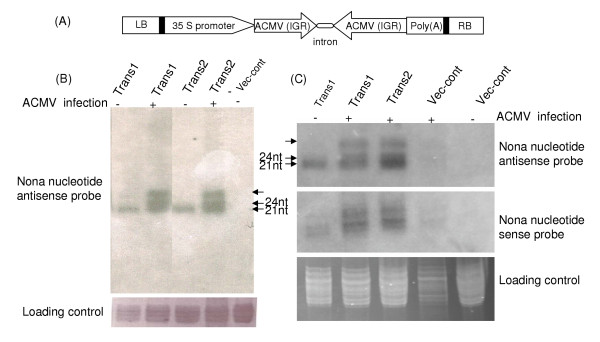
**Small RNA expression in ACMV-infected and non-infected transgenic and vector control plants**. **(A) **Schematic diagram of the binary construct used for plant transformation. LB, left border. RB, right border **(B, C) **Northern hybridization showing various siRNA size classes, two lower arrows indicate (known) approximate sizes and upper arrow indicate higher molecular weight size.

A transgene consisting of the promoter region of ACMV DNA A was designed to produce double stranded RNA (Figure 1A). A similar construct was shown to reduce the accumulation of Vigna mungo yellow mosaic geminivirus (VMYMV) in transient transfection system [7]. The *Nicotiana benthamiana *plants were transformed by *Agrobacteium*-mediated gene transfer. Resulting transgenic lines were infected with ACMV and tested for siRNA expression. Non-infected transgenic plants showed two classes of siRNAs, a less abundant 24–25 nt and more abundant 21–22 nt size class. However ACMV-infected transgenic lines showed small RNAs of approximate sizes, 21–22, 24–25 nt and also higher molecular weight siRNA size class, with equally higher expression intensities (Figure 1B–C).

**Figure 2:**
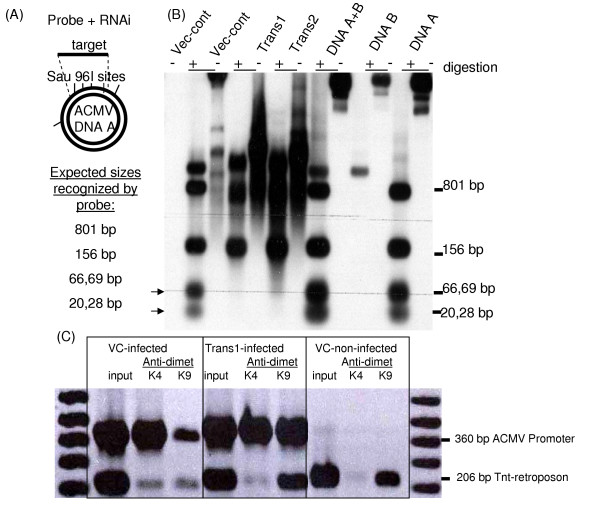
**Small RNA-directed DNA and histone methylation**. **(A) **Schematic representation of *Sau*96 I restriction sites in ACMV DNA A promoter region and expected sizes recognized by this probe **(B) **Southern hybridization showing fragments of ACMV generated by *Sau*96 I, arrows indicate fragments generated in vector control and protected in siRNA producing plants **(C) **ChIP duplex PCR analysis using ACMV DNA A promoter and Tnt-retroposon specific primers, sizes are indicated. K4, anti-dimethylated histone H3 lysine 4. K9, anti-dimethylated histone H3 lysine 9. VC, vector control.

I analyzed the molecular effects of these abundant small RNAs on ACMV genome in transgenic and vector control plants. The 21–22 nt siRNA class has been implicated in virus RNA and transgene mRNA degradation, whereas the longer size class 24–25 nt, in directing retroelement DNA methylation [2]. To test the methylation status of episomal DNA virus, the total genomic DNA from ACMV-infected transgenic and vector control plants was digested with methylation sensitive enzyme *Sau *96I. Southern hybridization was performed by using an ACMV DNA A promoter sequence specific probe. Analysis showed that at least three to four sites in the ACMV DNA A promoter region were protected from digestion only in siRNA expressing lines but not in vector control plant (Figure 2A–B). The sites protected by methylation flank the Nona-nucleotide sequence (which is the origin of replication or *ori*) in ACMV DNA A intergenic region as the same sequence was used to probe siRNAs.

The 24–25 nt siRNAs class along with *Argonaute-4 *has also been shown for retroelement silencing through histone H3 lysine 9 (H3K9) methylation in Arabidopsis [10]. The geminiviruses exist as minichromosomes in plant cells [5]. The histone methylation patterns of ACMV minichromosomes were determined by chromatin immunoprecipitation using anti-dimethyl histone H3 lysine 4 (H3K4) and anti-dimethyl histone H3 lysine 9 (H3K9). The ChIP PCR amplified an equal amount of ACMV DNA A promoter region fragment in the H3K4 immunprecipiates from both transgenic and vector control plants. However the enrichment of the same fragment in the H3K9 immunprecipiate was lower from the vector control plant compared to the small RNA producing transgenic plants (Figure 2C).

The presence of three small RNA species in these transgenic plants upon ACMV infection raises the possibility that RNAi might operate at three levels during DNA virus infection i.e. the mRNAs transcribed from geminivirus genome are subject to degradation by 21–22 nt small RNAs [6]. The geminiviral genomic DNA seems to be subject of RNA dependent DNA methylation (RdDM) by 24–25 nt small RNAs. However the observation showing an additional higher molecular weight siRNA class in transgenic plants might be similar to those 28 nt siRNAs observed in *Tetrahymena *[11]. And the fact that a micrococcal nuclease homologue also co-purifies with the RNAi effector complexes [12], raises further question whether the plants also possess small RNA directed DNA elimination mechanism in order to defend themselves from DNA viruses.

## Competing interests

The author(s) declares that they have no competing interests.

## Authors' contributions

AMD carried out all experimental work and drafted the manuscript.
